# Fluence-Dependent Changes in Surface Wettability and Conductivity of Ion-Irradiated Carbon-Based Foils

**DOI:** 10.3390/polym18040453

**Published:** 2026-02-11

**Authors:** Romana Mikšová, Petr Malinský, Eva Štěpanovská, Josef Novák, Petr Aubrecht, Vlastimil Mazánek, Anna Macková

**Affiliations:** 1 Nuclear Physics Institute CAS, Hlavni 130, 250 68 Husinec-Rez, Czech Republic; malinsky@ujf.cas.cz (P.M.); stepanovska@ujf.cas.cz (E.Š.); j.novak@ujf.cas.cz (J.N.); mackova@ujf.cas.cz (A.M.); 2 Faculty of Science, Jan Evangelista Purkyně University, Pasteurova 3632/15, 400 96 Usti nad Labem, Czech Republic; 3 Centre for Nanomaterials and Biotechnology, Faculty of Science, Jan Evangelista Purkyně University, Pasteurova 3632/15, 400 96 Usti nad Labem, Czech Republic; petr.aubrecht@ujep.cz; 4 Department of Inorganic Chemistry, University of Chemistry and Technology, Prague 6, 166 28 Prague, Czech Republic; vlastimil.mazanek@vscht.cz

**Keywords:** graphene oxide, cyclic olefin copolymer, gold ion implantation, electrical and surface properties

## Abstract

The surface properties and electrical behavior of carbon-based materials can be effectively modified by energetic ion irradiation. In the present study, graphene oxide (GO) and cyclic olefin copolymer foils (COC, Topas 112 and 011, respectively) were irradiated with 1 MeV Au ions using a 3 MV Tandetron accelerator at fluences of 1 × 10^14^, 1 × 10^15^, and 2.5 × 10^15^ cm^−2^. The irradiation induced systematic modifications in surface chemistry, morphology, wettability, and electrical properties. Composition changes were investigated using Rutherford backscattering spectrometry (RBS) and elastic recoil detection analysis (ERDA), while surface morphology and roughness were characterized by atomic force microscopy (AFM). This revealed a clear fluence-dependent evolution of nanoscale topography. The vibrational characteristics were assessed through Raman spectroscopy, and the chemical composition of the surface layers was analyzed by X-ray photoelectron spectroscopy (XPS). The surface wettability was evaluated by static contact angle measurements, and surface free energy was determined using the Owens–Wendt–Rabel–Kaelble (OWRK) method. These measurements showed a consistent decrease in water contact angle and an increase in surface free energy with increasing ion fluence in the COC substrates, whereas GO exhibited a distinct response. Electrical characterization demonstrated a pronounced fluence-dependent decrease in sheet resistivity in polymers. The results show that 1 MeV Au ion irradiation enables systematic and fluence-dependent modification of both surface and electrical properties.

## 1. Introduction

Polymeric and carbon-based thin foils are widely employed in modern optoelectronics [1], biosensing [2], and flexible devices [3]. Polymers are very well applied in tissue engineering due to a wide range of physical, mechanical, and chemical properties, cytocompatibility, microstructure, and biodegradability [4,5]. COC, including Topas 112 and Topas 011 foils, are chemically inert, optically transparent, and mechanically robust, but their intrinsically insulating nature limits their direct use in electronic applications without surface modification [6,7]. GO has attracted significant attention because of its tunable electrical conductivity, large surface area, and chemical versatility, which can be adjusted through reduction or functionalization processes [8].

Surface and bulk properties of these materials can be systematically changed using external treatments, such as chemical functionalization [9], plasma exposure [7,10,11], or energetic ion irradiation [12], or doping [6,13]. High-energy ion irradiation provides a unique pathway to simultaneously modify surface chemistry, morphology, and electrical properties in a controlled manner. Irradiation with MeV-range ions can induce a variety of physical and chemical processes, including chain scission, cross-linking, local densification, and sputtering, which, in turn, influence surface roughness, polarity, and electronic conductivity [6,8]. Ion irradiation can convert insulating properties’ polymer regions into partially conductive domains through mechanisms such as carbonization or formation of sp^2^-rich networks, while also altering wettability via the introduction of polar functional groups [6,8].

Previous studies have demonstrated that COC foils undergo significant surface and subsurface modifications under high-energy irradiation, which can gradually adjust surface energy and electrical properties [6,7]. GO responds sensitively to energetic ion exposure, with partial reduction and reorganization of the carbon lattice resulting in enhanced electrical conductivity and modified surface chemistry [8].

A detailed understanding of the interplay between ion fluence, nanoscale morphology, and electrical behavior is crucial for the design of functional materials. Surface roughness, for instance, can influence not only wettability [14] but also charge transport [15] and adhesion characteristics [16], while chemical modifications can create new active sites or conductive pathways. Therefore, characterizing both surface and bulk responses to controlled-ion irradiation is important to optimize performance in electronic, optoelectronic, and flexible device contexts.

Despite the reported potential of irradiation, a clear challenge remains in achieving a synergistic balance between surface hydrophilicity and electrical conductivity, two parameters that are often at odds during polymer carbonization. It is hypothesized that by selecting specific ion fluences, it is possible to trigger a dual-action modification. At these fluences, the energy deposition is sufficient to induce partial carbonization for charge transport, while simultaneously creating oxygen-rich radical sites that react with the atmosphere to form polar functional groups. This dual modification is particularly critical for resistive-type humidity sensors, where the surface must be both hydrophilic (to adsorb water molecules) and sufficiently conductive (to translate adsorption into a measurable electrical signal). The objective of this study is to determine which architectural design offers superior stability and sensitivity for environmental monitoring applications. To this end, a comparative analysis is conducted between the 2D lattice of GO and the 3D structure of COC. This study does not merely characterize surface changes but seeks to identify the optimal irradiation parameters where Topas and GO foils transition from passive substrates into functional components with tunable wettability and enhanced electrical conductivity for flexible electronics.

This work offers the relationship between irradiation parameters, surface and compositional evolution, and electrical properties, offering insights into the controlled engineering of carbon-based foils for advanced functional applications. While GO and COC represent different structural classes of carbon-based materials, they both serve as promising candidates for flexible sensing platforms. Understanding their divergent responses to ion-beam irradiation is crucial for integrating them into multi-material sensor devices. In the case of COC, the primary motivation for this study is to determine how varying the norbornene content influences the fundamental competition between ion-induced chain scission and cross-linking. Higher norbornene content (NB) results in COCs with higher glass transition temperatures (T_g_), such as Topas 011, which has a lower NB content and T_g_ (78 °C). The lower thermal stability of Topas 011 makes it more susceptible to ion-beam-induced surface softening and reorganization (smoothing) during irradiation, as local energy deposition can more easily exceed its glass transition threshold. The present study investigates the effects of 1 MeV Au ion irradiation on COC and GO foils over a range of fluences (1 × 10^14^, 1 × 10^15^, and 2.5 × 10^15^ cm^−2^). We employ a multifaceted approach that integrates compositional analysis using Rutherford backscattering spectrometry (RBS) and elastic recoil detection analysis (ERDA) with surface characterization through atomic force microscopy (AFM) and wettability measurements, as well as X-ray photoelectron spectroscopy (XPS) and Raman spectroscopy. Electrical behavior is evaluated through sheet resistivity measurements, providing a comprehensive view of irradiation-induced modifications.

## 2. Materials and Methods

### 2.1. Materials

The Topas substrates were used for ion irradiation and subsequent testing of functional properties: mcs-foil 112 with a thickness of 175 µm and a glass transition temperature (T_g_) of 142 °C, corresponding to Topas grade 6015 with a norbornene content of 52–57%; and mcs-foil 011 with a thickness of 140 µm and a T_g_ of 78 °C, corresponding to Topas grade 8007 with a norbornene content of 35–45%. Both substrates were supplied by ChipShop (Jena, Germany) [17].

GO was synthesized by permanganate oxidation of graphite [18]. Three grams of graphite were mixed with H_2_SO_4_/H_3_PO_4_ (360/40 mL), followed by 18 g KMnO_4_ and heating at 50 °C for 12 h. The reaction was quenched with ice (400 g) and H_2_O_2_ (20 mL), and GO was collected by centrifugation. GO foils were prepared by suction filtration using a poly carbonate membrane (Nucleopore 0.45 μm/90 mm diameter) and 10 mL of GO aqueous suspension with a concentration of 6.7 mg mL^−1^.

### 2.2. Analytical Methods

RBS and ERDA were employed for the compositional study of the foils before and after the ion irradiation with the ion fluences of 1 × 10^14^, 1 × 10^15^, and 2.5 × 10^15^ cm^−2^. The RBS spectra were collected using a beam of 2.0 MeV He^+^ ions (in the remainder of the article, the ion will be referred to without specifying its charge state). An Ultra-Ortec PIPS detector recorded the He ions backscattered at a laboratory scattering angle of 170°. The ERDA spectra were measured using 2 MeV He ions, with the primary beam coming at an angle of 75° with respect to the substrate surface normal and with hydrogen atoms recoiled at a scattering angle of 30° registered with the PIPS detector covered by a 12 µm Mylar foil. To reduce the effects of the sample degradation during the RBS/ERDA, several spectra were measured on different beam spots on the sample surface, and the final spectrum was obtained by summing the individual spectra. RBS/ERDA spectra were evaluated by the SIMNRA code [19].

Surface morphology and roughness were analyzed by AFM (XploRA PLUS, Horiba Jobin Yvon, Palaiseau, France) in tapping mode using HQ:NSC14 probes with an aluminum reflective coating (spring constant 5 N·m^−1^) under ambient conditions at room temperature. The data were analyzed using the free software Gwyddion version 2.69. Raman spectroscopy was also carried out with the same system using a confocal lens.

Raman microscope (XploRA PLUS, Horiba Jobin Yvon, Palaiseau, France) equipped with a 638 nm diode laser excitation source. Measurements were performed under ambient conditions at room temperature, focused through a 100× objective with a high aperture, with an acquisition time of 15 s and an accumulation number of 3. The laser power was kept below 1.5 mW on the sample, and the signal was dispersed by a grating 1800 L/mm.

The electronic structure and chemical composition of the surface layers were analyzed by XPS, with an Omicron Nanotechnology ESCAProbeP spectrometer, London, England. The X-ray monochromatic source at 1486.7 eV was used, and XPS spectra were evaluated using CasaXPS 2.3.26 software.

Electrical properties of the pristine and irradiated foils were studied by the standard 2-point method using the Keithley 6221 current source and Keithley 2128A nanovoltmeter (Tektronix, Solon, OH, USA). For the electrical resistance measurement, Au contacts (50 nm thick) with a relative distance of 1 mm were sputtered on the surface of the foils. For more details, see our previous study [20].

Surface wettability was evaluated by static contact angle measurements using the sessile drop method (DSA30, Krüss, Hamburg, Germany) at 22–24 °C (±0.1°). A 2 µL drop of deionized water or diiodomethane was deposited at 2 µL min^−1^, and contact angles were recorded after 10 s of stabilization. Each sample was measured four times. Contact angles were calculated using Advance software with an Ellipse (tangent-1) fit and automatic or manual baseline.

SFE was calculated in Advance software employing the OWRK method from the contact angles of two liquids: water as polar and diiodomethane (CH_2_I_2_) as non-polar. This model considers the surface tension from the viewpoint of the polar and dispersive components [21,22]. In total, eight values from each liquid were used to calculate the surface free energy.

### 2.3. Ion-Beam Implantation and Simulation

Topas 112/011 and GO thin foils were irradiated with 1 MeV Au ions in the implantation chamber of the 3 MV Tandetron MC-4130 accelerator at the Nuclear Physics Institute of the Czech Academy of Sciences. The Au ions were generated in a source of negative ions by cesium sputtering from a solid gold target. The irradiation was carried out at normal incidence (0° relative to the surface normal) within an implantation chamber evacuated to a base pressure of approximately 6.9 × 10^−6^ mbar. The ion current density was maintained between 7 and 10 nA cm^−2^, with applied ion fluences of 1 × 10^14^, 1 × 10^15^, and 2.5 × 10^15^ cm^−2^.

The projected ranges (R_P_) and the range straggling (ΔR_P_) extracted from the measured depth profiles are presented as well and compared with the corresponding SRIM [23]. The calculations were performed using a full cascade Monte Carlo simulation, with the initial material composition taken from RBS analysis of the real samples. Energy loss of incident ions occurs through electronic and nuclear stopping, which together drive irradiation-induced substrate modifications. Electronic stopping primarily induces ionization and electronic excitation, whereas nuclear stopping causes atomic displacements and bond rupture [24]. Both processes degrade the host matrix via chain scission, free-radical cross-linking, and bond conjugation. Dominant electronic stopping favors dehydrogenation and formation of low-mass fragments, while nuclear stopping promotes degassing and carbonization [25]. Electronic (S_e_) and nuclear stopping (S_n_) powers, as well as R_p_ and (ΔR_P_) for Topas 112/011, and GO are shown in Table 1.

For all investigated materials, the nuclear stopping power dominates over electronic stopping, as reflected by S_e_/S_n_ ratios ranging from 0.61 to 0.77. The highest relative contribution of electronic stopping is observed for Topas 112 and Topas 011, indicating that interactions between ions and electrons play a major role in energy dissipation within these cyclic olefin copolymers. In contrast, GO exhibits the lowest S_e_/S_n_ ratio, suggesting a significantly stronger contribution to nuclear stopping. This is consistent with the higher atomic density and carbon-rich structure of GO, as evidenced by its higher S_n_ value (183 eV/nm) compared to Topas (160 eV/nm).

The projected ion ranges R_p_ decrease from Topas (421–422 nm) to GO (358 nm), reflecting differences in material density and elemental composition. The longitudinal straggling ΔR_p_ remains similar for Topas and GO (51 nm).

## 3. Results

### 3.1. RBS and ERDA, and Au Depth Profiles

**Figure 1:**
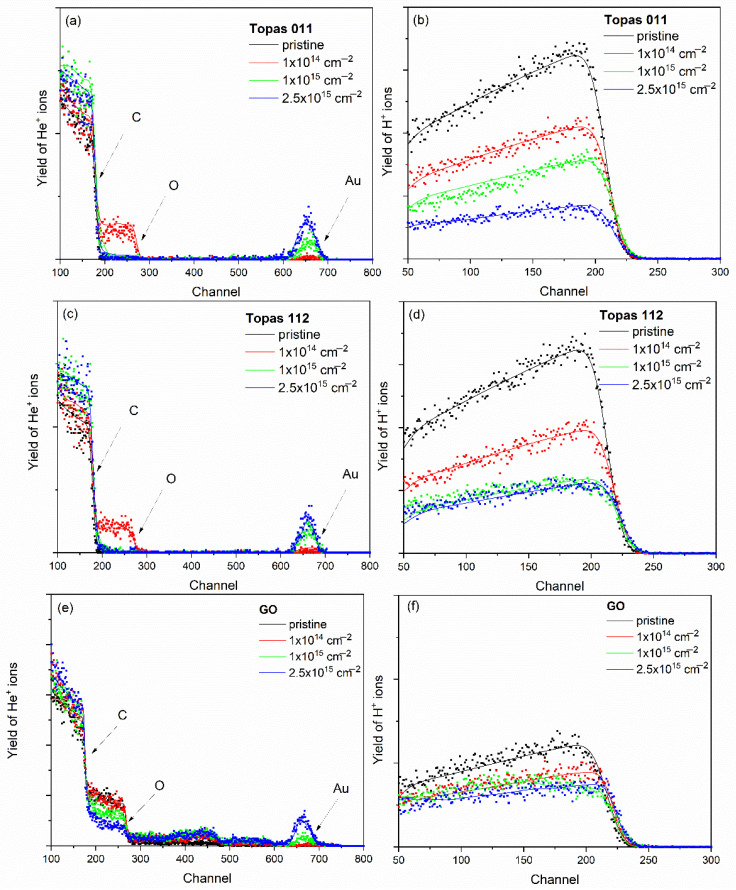
The RBS experimental spectra (symbols) together with the corresponding SIMNRA-simulated spectra (solid lines) for pristine and 1 MeV Au-ion-irradiated Topas 011 (**a**), Topas 112 (**c**), and GO (**e**). The experimental ERDA spectra (symbols) and the corresponding SIMNRA simulations (solid lines) for the Topas 011 (**b**), Topas 112 (**d**), and GO (**f**) irradiated at fluences ranging from 1 × 10^14^ to 2.5 × 10^15^ cm^−2^.

The elemental composition of pristine and ion-irradiated samples was analyzed using ion-beam spectroscopic techniques, namely RBS and ERDA, employing 2 MeV He ions. For polymers and graphene oxide, the effective information depth of RBS at this beam energy is approximately 1 µm, while ERDA provides a shallower probing depth of about 0.5 µm, making the two techniques complementary for compositional analysis [20]. RBS and ERDA spectra for pristine Topas 112/011 and GO samples irradiated with 1 MeV Au ions at fluences of 1 × 10^14^, 1 × 10^15^, and 2.5 × 10^15^ cm^−2^ are shown in Figure 1. The experimental spectrum was evaluated using SIMNRA simulations, enabling quantitative determination of elemental composition and depth distributions.

**Figure 2:**
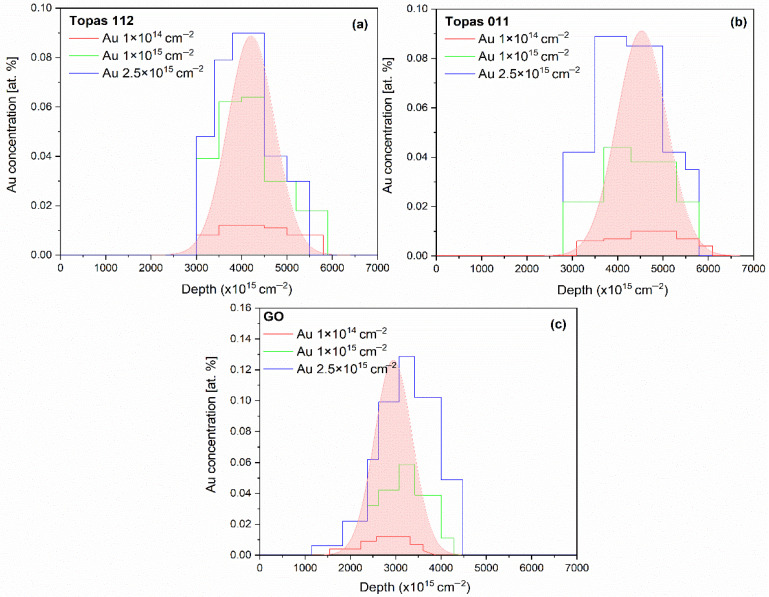
The experimental depth profiles of Au atoms in irradiated Topas 112/011 (**a**,**b**) and GO (**c**) foils irradiated by 1 MeV Au ions with fluences in the range of 1 × 10^14^–2.5 × 10^15^ cm^−2^. Au depth profile simulated using SRIM is shown also (pink distribution).

In addition, Au depth profiles were extracted from the 2 MeV RBS spectra and are presented in Figure 2, providing insight into the implantation depth and distribution of Au ions within the polymer matrix.

Figure 2a,b shows the experimental Au depth distributions in irradiated Topas 112/011 foils, respectively. For the lowest ion fluences, the Au concentration profile exhibits a well-defined peak located at a depth corresponding to the projected range of 1 MeV Au ions, with a relatively symmetric distribution around the maximum. As the ion fluence increases, the peak intensity increases proportionally, accompanied by a gradual broadening of the profile, indicating increased ion straggling and accumulation effects. In the case of Topas 011 (Figure 2b), noticeable changes in the Au depth distribution are observed. The peak position shows a slight shift toward the surface, and the profiles become broader and more asymmetric, particularly at higher fluences. These effects suggest irradiation-induced modifications of the polymer matrix, including changes in density, enhanced damage accumulation, and possible surface recession due to sputtering. For Topas 112/011, the experimentally measured depth profiles are compared with SRIM simulations, which predict a narrower distribution. The deviations between experiment and simulation become more pronounced with increasing fluence, reflecting the limitations of static SRIM calculations in capturing fluence-dependent structural changes in polymer targets.

Figure 2c shows the experimental depth profiles of Au implanted into GO foils under identical irradiation conditions. Compared to polymer samples, Au in GO exhibits a shallower projected range and a pronounced asymmetry with an extended tail toward greater depths, reflecting GO’s higher density, layered structure, and compositional heterogeneity. Increasing ion fluence leads to higher Au concentrations and broadening of the profiles, indicative of enhanced defect formation, ion-beam-induced mixing, and local structural rearrangements. At the highest fluences, the profiles shift deeper and deviate from Gaussian behavior, developing pronounced shoulders at both shallow and deep regions, consistent with Au permeation previously observed in bulk GO and other materials [26]. Comparison with SRIM simulations reveals systematic discrepancies, with experimental profiles being broader and shifted relative to the simulated distributions.

Table 2 summarizes the elemental composition of pristine and Au-ion-irradiated Topas 112/011 and GO foils as determined by combined RBS and ERDA analyses. These techniques provide complementary information on the concentration of all elements (C, O, N, and H), enabling a comprehensive evaluation of irradiation-induced compositional changes.

For pristine Topas 112 and Topas 011 foils, elemental compositions align with their nominal polymer structures, dominated by carbon and hydrogen. Irradiation with 1 MeV Au ions induces pronounced, fluence-dependent compositional changes. In both polymers, increasing fluence systematically raises the carbon content while reducing hydrogen, reflected in a monotonic increase in the C/H ratio, indicative of progressive hydrogen depletion. This behavior is characteristic of ion-beam-induced dehydrogenation, driven by hydrogen’s low displacement energy and high mobility.

Oxygen exhibits non-monotonic evolution: initially increasing at low fluences, then stabilizing or slightly decreasing at higher fluences, likely due to competing irradiation-induced oxidation from residual atmosphere and oxygen loss via bond scission and sputtering. The increasing C/O ratio with fluence supports carbon enrichment of the polymer matrix, associated with chain scission, cross-linking, and formation of carbon-rich structures. These trends are consistent with structural transformations inferred from depth-profile and spectroscopic analyses.

Pristine GO is different, with higher oxygen content and a larger C/O ratio reflecting its oxidized layered structure. Au ion irradiation moderately increases carbon while strongly decreasing oxygen, resulting in a substantial rise in the C/O ratio, indicative of irradiation-induced reduction via preferential oxygen removal. The hydrogen content in pristine GO was initially low (13 at.%) and decreased to 7.8 at.% at the highest irradiation fluence, suggesting that while hydrogen loss occurs, hydrogen-related processes are less dominant in GO than in the copolymers. The observed compositional evolution correlates with defect generation, partial restoration of sp^2^ carbon networks, and structural rearrangements. At the highest fluence (2.5 × 10^15^ cm^−2^), severe structural damage and partial material removal hinder accurate RBS/ERDA quantification.

RBS and ERDA reveal material-dependent responses to Au ion irradiation, where Topas polymers undergo dehydrogenation and carbon enrichment linked to degradation and cross-linking, while GO experiences pronounced oxygen depletion consistent with reduction and structural transformation. The oxygen increase in polymers may also reflect oxidation after irradiation. The pristine Topas structure is dominated by C–C and C–H bonds [27], which primarily induce electronic stopping-driven bond scission [20,25]. Hydrogen loss results from macromolecular cleavage and free-radical formation, while oxygen incorporation enhances hydrophilicity of the irradiated polymers [27].

### 3.2. Surface Morphology

**Figure 3:**
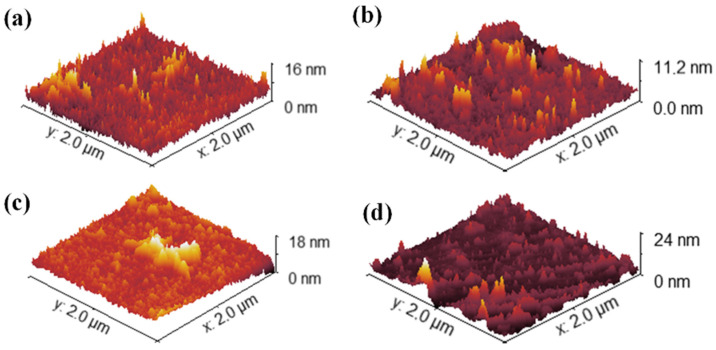
Surface morphology measured by AFM. Representative 2D images (2 × 2 µm^2^) of pristine TOPAS 011 foils (**a**) and those modified by 1 MeV Au ions with fluences of 1 × 10^14^ (**b**), 1 × 10^15^ (**c**), and 2.5 × 10^15^ cm^−2^ (**d**).

AFM was employed to evaluate the evolution of surface topography in Topas 112, Topas 011, and GO substrates subjected to 1 MeV Au ion irradiation at fluences of 1 × 10^14^, 1 × 10^15^, and 2.5 × 10^15^ cm^−2^. For all materials, both the arithmetic roughness (R_a_) and the root-mean-square roughness (RMS) were extracted from 2 × 2 μm^2^ scans to quantify irradiation-induced morphological modifications (see Figure 3 for Topas 011) and are collected in Table 3. The mean value and standard deviation of the selected quantity are calculated from the set of individual row values of each scan in the Gwyddion program.

For the unirradiated Topas 112 foils, the pristine surface exhibited a R_a_ of 1.20 nm and an RMS of 1.35 nm. Upon increasing ion fluence, both roughness metrics decreased markedly, achieving minimum values of 0.30 nm (R_a_) and 0.39 nm (RMS) at 1 × 10^14^ cm^−2^, and remaining below pristine levels at higher fluences (R_a_ ≈ 0.71–0.75 nm, RMS ≈ 1.06–1.07 nm), indicating smoothing of the surface likely due to ion-induced densification and defect annealing effects. Similarly, Topas 011 foils demonstrated a reduction in Ra from 1.16 nm to as low as 0.72 nm, with corresponding RMS values decreasing from 1.45 nm to 1.03 nm at the lowest studied fluence.

GO foils had substantially higher initial roughness (Ra ≈ 20 nm, RMS ≈ 24 nm) that increased slightly at 1 × 10^14^ cm^−2^ (Ra ≈ 26–24 nm, RMS ≈ 32–29 nm) before decreasing at the highest fluence (Ra ≈ 16.5 nm, RMS ≈ 20.0 nm).

### 3.3. Raman Measurement

**Figure 4:**
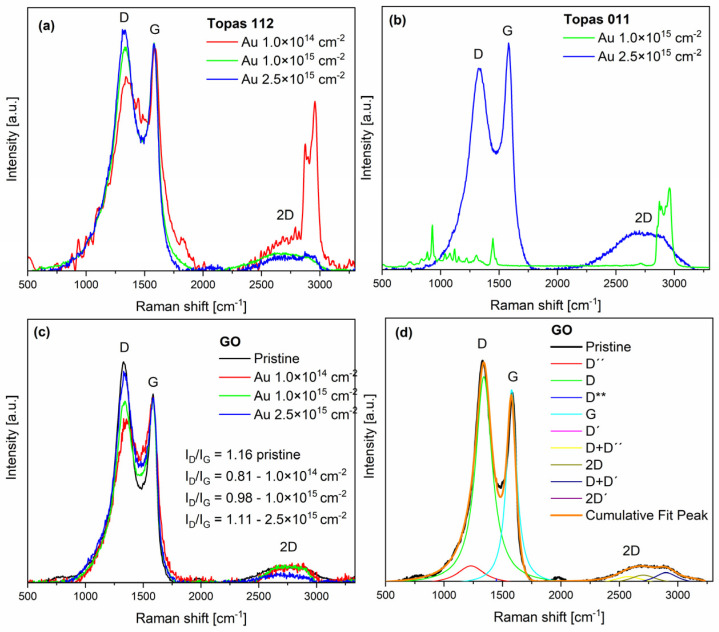
Raman spectra of Topas 112 (**a**), Topas 011 (**b**), and GO (**c**) after 1 MeV Au ion irradiation at fluences from 1 × 10^14^ to 2.5 × 10^15^ cm^−2^, recorded with a 632 nm excitation wavelength. Deconvolution of the pristine GO (**d**).

Raman spectroscopy provides a detailed analysis of structural changes caused by gold beam irradiation. The spectra exhibit the characteristic features of sp^2^-hybridized carbon materials, notably the D, G, and 2D bands, along with several additional fitted peaks D″, D**, D + D″, D′, D + D′, and 2D′. These peaks were deconvoluted through multi-peak fitting (Lorentzian function was used for all peaks in this purpose [28], see Figure 4d), providing deeper insight into the disorder, stacking order, and complexity of the carbon structures.

The D″ band (~1230 cm^−1^) is a minor feature typical of amorphous carbon. The D peak (~1350 cm^−1^) arises from breathing modes of six-membered rings and requires defects for activation [29]. The D** feature (~1450 cm^−1^) is associated with amorphous carbon or edge-related vibrations [30]. The G peak (~1580 cm^−1^) corresponds to the E_2g_ high-frequency phonon and is present in all carbon materials containing sp^2^ bonding [31]. A second peak is found very close to the G band, but slightly upshifted (about 1620 cm^−1^). It is a D′ peak due to an intravalley double-resonance process, linking points within the same cone near K (or K′) [32].

At higher Raman shifts, second-order and combination modes appear. The D+D′′ band (~2580 cm^−1^) represents a second-order combination mode of graphitic carbon, providing a characteristic signature of sp^2^ hybridized structures and their disorder. The 2D band (~2700 cm^−1^) is the overtone of the D peak, arising from second-order zone-boundary phonons. These phonons are Raman-inactive in first-order spectra of defect-free graphite, appearing only through double-resonance processes [33].

The D+D′ band (2850–3000 cm^−1^) arises from a two-phonon defect-assisted scattering process, involving one phonon near the K point (D) and another near the Γ point (D′). The presence of a defect enables the necessary momentum relaxation, allowing both phonons to participate in the double-resonance process [32]. The band center frequency differs from the simple sum of the positions of D and D′ peaks. The 2D′ band (~3200 cm^−1^) is the overtone of the D′. Both the 2D and 2D′ bands arise from defect-independent double-resonance processes involving two phonons with opposite momenta, which satisfy momentum conservation, making these overtones intrinsic features of sp^2^ carbon systems [32]. The D band is defect-activated, with I_D_/I_G_ reflecting structural disorder. The 2D band, a second-order overtone, is sensitive to graphene thickness and stacking. The I_2D_/I_G_ ratio is commonly used to estimate the number of graphene layers: monolayer graphene exhibits I_2D_/I_G_ > 1.4, bilayer graphene ranges from 0.75 to 1.4, and multilayer graphene is below 0.75 [34].

The pristine Topas 112/011 Raman spectrum, as well as Topas 011 irradiated with low fluence, is overwhelmed by a high fluorescence background. The Raman spectra of irradiated Topas 112 and 011 show several peaks originating from polymeric structures (see Figure 4a for Au 1 × 10^14^ cm^−2^ and Figure 4b for Au 1 × 10^15^ cm^−2^). The strong bands observed at 750, 890, and 930 cm^−1^ correspond to norbornene units in COC. In the COC spectrum, the bands at 750 and 930 cm^−1^ are assigned to ring vibrational modes of the COC molecules, while the ring breathing mode is observed at 889 cm^−1^ in the Raman spectrum. The Raman peaks at 1225 and 1311 cm^−1^ are attributed to the twisting modes of the CH_2_ groups in COC. C–H symmetric stretching is observed between 2800 and 3100 cm^−1^ [35,36]. As evidenced by the Raman spectra of Topas 112 (Figure 4a), the intense and well-defined C–H stretching modes in this region, typical for the polymer, undergo a dramatic reduction in intensity upon Au ion irradiation. This systematic quenching of the C–H signals directly tracks the ion-induced dehydrogenation of the polymer matrix. The simultaneous depletion of the aliphatic C–H peaks and the emergence of broad D and G bands further confirm the structural transition from a saturated hydrocarbon chain to a carbonized, sp^2^-rich network.

The most prominent peaks are also the D and G peaks in all Raman spectra of GO (Figure 4c). The decrease in D-peak intensity and the increase in the 1450 cm^−1^ peak after gold irradiation become more pronounced with lower ion fluence. The reduction in the D peak reflects the loss of epoxide and hydroxyl groups in GO, while the diminishing multiple features between 2400 and 3200 cm^−1^ indicate the growth of sp^2^ domains [20,37], which was also reflected by RBS. After irradiation with 1 × 10^15^ and 2.5 × 10^15^ cm^−2^, the D peak increased again. The D peak growth is caused by the formation of non-six-carbon rings [38,39].

In pristine GO, the I_D_/I_G_ ratio is 1.16, reflecting a high level of disorder due to oxygen-containing functional groups. After gold irradiation, the I_D_/I_G_ ratio first decreases to 0.81 at a low fluence of 1 × 10^14^ cm^−2^, indicating that initial irradiation partially removes epoxide and hydroxyl groups, restoring sp^2^ domains and reducing disorder. At higher fluences, I_D_/I_G_ increases to 0.98 (1 × 10^15^ cm^−2^) and 1.11 (2.5 × 10^15^ cm^−2^) as continued ion impacts create new defects, increasing lattice disorder. The 2D peak steadily decreases with fluence, reflecting the progressive disruption of long-range sp^2^ order despite the partial reduction in oxygen groups.

### 3.4. XPS

**Figure 5:**
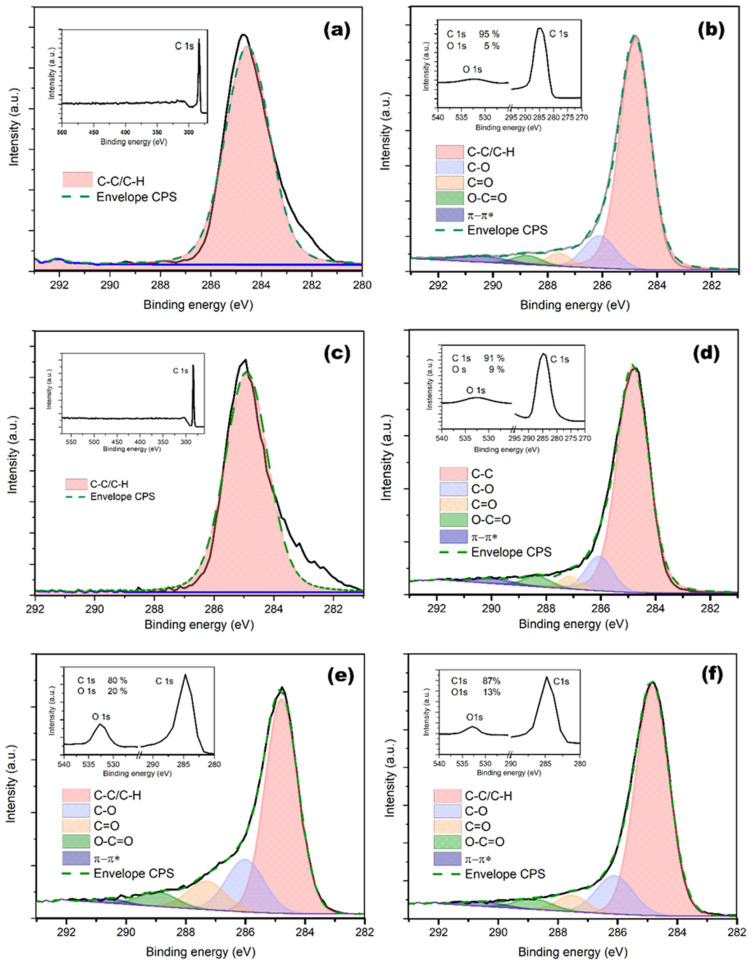
XPS spectra of pristine and irradiated (1 MeV Au ions, 2.5 × 10^15^ cm^−2^) Topas 112 (**a**,**b**), Topas 011 (**c**,**d**), and GO (**e**,**f**). XPS survey spectra are in the inset.

For the evaluation of the carbon-to-oxygen (C/O) ratios from the survey spectra, relative sensitivity factors were used and are presented in Figure 5 and summarized in Table 4. The results exhibit a good correlation with the RBS/ERDA measurements. The measured spectrum exhibits a slight asymmetry and broadening in the peak, particularly toward lower binding energies. Given the absence of a significant oxygen component in the corresponding O 1s spectrum, these features cannot be unambiguously attributed to oxygenated carbon bonds. Instead, they are interpreted as line-shape artifacts arising primarily from differential surface charging on the insulating COC polymer, which produces a characteristic low-binding-energy tail (~282–284 eV) due to non-uniform charge buildup [40,41].

In pristine polymers, both Topas 011 and Topas 112 exhibited a purely hydrocarbon character, with the C–C/C–H component accounting for ~100% of the C 1s signal, which is consistent with their stoichiometric structure. Following irradiation at the lowest fluence (1 × 10^14^ cm^−2^), pronounced surface oxidation was observed for both polymers. The C–C/C–H contribution decreased to approximately 72–74%, accompanied by a substantial increase in oxygen-containing functional groups. This oxidation was dominated by C–O species (≈13–17%), with smaller contributions from C=O and O–C=O groups. These changes are attributed to the formation of ion-induced reactive sites, such as radicals and defects, which subsequently undergo post-irradiation oxidation upon exposure to ambient atmosphere. As the fluence increased to 1 × 10^15^ cm^−2^, a transition in the modification mechanism became apparent. In Topas 112, the relative concentration of C–O groups decreased, while the C=O component increased to approximately 7%, indicating a deeper structural reorganization and the formation of more stable carbonyl configurations. In contrast, Topas 011 exhibited the emergence of a π–π* shake-up feature (~2%), signaling the onset of surface carbonization and the formation of conjugated sp^2^-rich domains. At the highest fluence (2.5 × 10^15^ cm^−2^), both polymers showed a trend toward a more carbon-like surface composition. The C–C/C–H component stabilized at elevated levels (~79–81%), while the relative contribution of oxygen-containing functional groups further decreased. The persistence of the π–π* transition supports the interpretation that high-fluence irradiation promotes cross-linking and partial carbonization of the near-surface region, which effectively suppresses the formation of stable oxygen-containing functionalities.

In contrast to the polymers, the pristine GO sample exhibited a strongly oxidized surface chemistry, with the C–C/C–H component accounting for approximately 66%, accompanied by substantial contributions from oxygen-containing functional groups, namely C–O (~17%), C=O (~9%), and O–C=O (~5%), which is characteristic of graphene oxide. Upon irradiation, GO underwent a predominantly reductive modification rather than further oxidation. Already at the lowest fluence (1 × 10^14^ cm^−2^), an increase in the C–C/C–H contribution to ~74% was observed, together with a reduction in all oxygen-containing components. With increasing fluence to 1 × 10^15^ cm^−2^, this trend became more pronounced, and the C–C/C–H content increased further to ~80%, indicating a more effective removal of oxygen functionalities and partial restoration of the sp^2^-hybridized carbon network. At the highest fluence (2.5 × 10^15^ cm^−2^), the surface composition stabilized, with the C–C/C–H contribution remaining at ~78% and oxygen-containing groups showing no further systematic decrease. This behavior suggests a balance between ion-induced reduction and carbonization of the GO surface and the re-oxidation of residual defect sites upon post-irradiation exposure to the ambient atmosphere.

### 3.5. Wettability and Surface Energy

**Figure 6:**
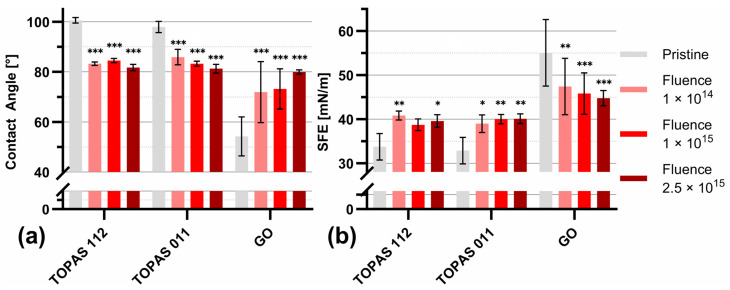
Surface wettability characterization of Topas 112 and Topas 011 foils modified by 1 MeV Au with fluences of 1 × 10^14^, 1 × 10^15^, and 2.5 × 10^15^ cm^−2^. Water contact angle measurements (**a**) and surface free energy (SFE) values (**b**) calculated using the OWRK method. Data are presented as mean ± SD (n = 8) and analyzed by two-way ANOVA comparing pristine and modified materials. Significance is indicated as * *p* ≤ 0.05, ** *p* ≤ 0.01, and *** *p* ≤ 0.001; unlabeled data are not statistically significant.

Figure 6 summarizes the evolution of the water contact angle (Figure 6a) and surface free energy (SFE, Figure 6b) for Topas 112, Topas 011, and GO foils subjected to ion irradiation at fluences of 1 × 10^14^, 1 × 10^15^, and 2.5 × 10^15^ cm^−2^. For all investigated materials, ion-beam treatment induces a systematic modification of the surface wettability. For Topas 112/011, the pristine samples exhibit contact angles of 100 ± 2° and 101 ± 2°, consistent with their intrinsically hydrophobic character. Upon irradiation, a gradual decrease in contact angle is observed, with the most pronounced reduction at the highest fluence.

Topas 112 and Topas 011 show statistically significant reductions across all fluence levels, with contact angle decreases of up to ~10° at 2.5 × 10^15^ cm^−2^. GO exhibits an opposite and more pronounced response, reflecting its higher chemical sensitivity to irradiation-driven structural rearrangements. GO is hydrophilic; during the production process, oxygen-containing functional groups such as -OH and -COOH are attached to the surface and edges of the graphene layers. After gold ion-beam irradiation, the surface of the GO is more hydrophobic.

The OWRK method was used to calculate SFE. The corresponding SFE values (Figure 6b) increase with fluence for Topas 112/011 compared to the pristine sample. The SFE of pristine TOPAS 112 and 011 was 32 ± 2 and 31 ± 1 mN m^−1^, respectively, classifying them as low-energy polymers, like PTFE, PDMS, and polypropylene, which typically exhibit SFE in the range of 18–30 mN m^−1^ [6]. The most notable enhancement is observed for Topas 011, where the SFE rises from ~31 mN m^−1^ (pristine) to nearly 40 mN m^−1^ (2.5 × 10^15^ cm^−2^).

The total SFE is the sum of its independent polar and dispersive components. Specifically, the dispersive component accounts for non-specific London dispersion forces, while the polar component represents specific interactions such as dipole–dipole, induction, and hydrogen bonding [42]. To elucidate the mechanisms driving the observed changes in wetting behavior, the distribution of these components is summarized in Table 5.

The results indicate that the increase in total SFE in the case of Topas 011/112 is predominantly driven by the polar component, which rises more significantly than its dispersive counterpart. This shift is attributed to the formation of polar species through a complex series of radiation-induced processes, including chain scission, branching, recombination, and post-irradiation oxidation [43]. The substantial increase in polarity is linked to the enrichment of the surface with oxygen-containing functional groups. These structures provide active sites for free radicals, which facilitate further oxidation upon exposure to air. The overall increase in SFE demonstrates that 1 MeV Au ion irradiation successfully functionalizes the surface of both Topas grades, transforming their naturally hydrophobic character into a significantly more hydrophilic state that is contrary to GO.

### 3.6. The Electrical Properties

**Figure 7:**
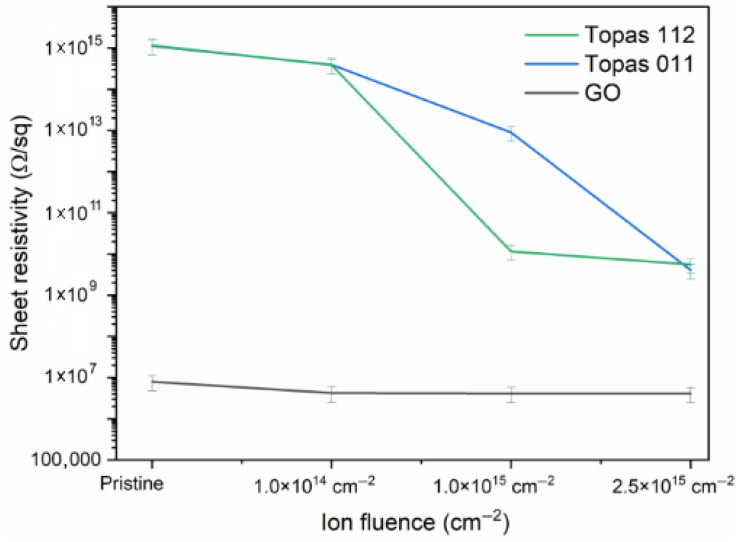
The sheet resistivity depends on the ion fluence of Topas 112, Topas 011, and GO.

Figure 7 shows the evolution of the sheet resistivity of Topas 112/011 and GO foils implanted with 1 MeV Au ions with different ion fluences. Upon ion implantation, all materials show a decrease in sheet resistivity, demonstrating that Au ion irradiation effectively enhances their electrical conductivity.

Topas 112 and Topas 011 exhibit higher initial resistivity and a more moderate conductivity enhancement compared to GO. Both polymeric materials show a monotonic decrease with increasing fluence, confirming that even chemically inert cyclic olefin copolymers undergo sufficient structural modification under 1 MeV Au ion bombardment to facilitate charge transport. Compared to Topas 112 and 011, GO exhibits a relatively monotonic behavior with a slight decrease in sheet resistivity after irradiation.

## 4. Discussion

For the polymeric substrates, the interaction is characterized by a high nuclear stopping component (S_n_). The observed monotonic increase in the C/H ratio for both Topas grades confirms progressive dehydrogenation, a typical phenomenon in ion-beam-induced degradation [25,44]. This process facilitates the transition from saturated hydrocarbons to carbonized, sp^2^-rich networks, evidenced by the formation of the Raman D and G bands.

The role of norbornene (NB) is particularly critical in defining the radiation stability of the COC matrix. Topas 011, which possesses a lower NB content and a significantly lower glass transition temperature (T_g_ = 78 °C), exhibited more pronounced surface reorganization compared to the more rigid Topas 112 (T_g_ = 142 °C). In contrast with this finding, GO undergoes a reductive process characterized by preferential oxygen depletion. At low fluences, this reduction partially restores sp^2^ domains, initially decreasing the I_D_/I_G_ ratio (see Figure 4c). As the fluence increases, ion impacts introduce a new regime of lattice disorder and defects, leading to a secondary increase in Raman D-peak intensity and structural amorphization.

A decrease in roughness was observed for Topas at low irradiation fluences. This phenomenon can be attributed to an ion-beam polishing effect, where the high energy density deposited by Au ions induces localized surface melting and increased molecular mobility. This allows for the redistribution of material from surface peaks to valleys before the surface is stabilized by a densified, cross-linked skin layer. Similar smoothing effects have been documented in other ion-irradiated polymers, such as PMMA, where competitive processes between chain scission and cross-linking initially favor a more uniform surface morphology [45].

The evolution of surface wettability functions as a macro-scale indicator of these chemical changes. For the Topas polymers, the consistent decrease in water contact angles and the rise in SFE are attributed to the formation of ion-induced reactive sites (radicals and defects) that undergo post-irradiation oxidation upon exposure to the atmosphere [17]. This effectively changes the naturally hydrophobic COC surfaces into more hydrophilic ones through the incorporation of polar oxygen-functional groups, such as C–O and C=O. In the case of GO, the situation is different, and GO transitions toward a more hydrophobic state. Pristine GO is already highly oxidized; therefore, ion bombardment acts primarily to remove existing hydrophilic groups, such as epoxide and hydroxyl groups, thereby reducing its surface polarity [46].

The significant reduction in sheet resistivity across all substrates indicates the effectiveness of Au ion irradiation in engineering conductive pathways. In Topas, this conductivity enhancement is a direct result of the carbonization process, which gives rise to the formation of conjugated sp^2^ domains. These domains play a pivotal role in facilitating charge transport within the polymer, which would otherwise be insulating. While the enhancement is more moderate in polymers compared to the already semi-conductive GO, the results confirm that 1 MeV Au ion irradiation enables systematic, fluence-dependent modification of electrical properties. The changes in the electrical conductivity after gold ion irradiation can be attributed to the decrease in oxygenation levels within GO, as proved by XPS and RBS measurements, and to the creation of excessive sp^2^ domains, as evidenced by XPS and Raman spectroscopy [8].

## 5. Conclusions

The application of ionizing radiation, such as that produced by the Au ion, has been demonstrated to induce material-dependent modifications in polymeric Topas (112 and 011) and GO foils. In the Topas 112/011 specimen, the effect of irradiation causes dehydrogenation, carbon enrichment, chain scission, cross-linking, and partial amorphization. These phenomena are evidenced by D and G Raman bands, surface smoothing, increased surface free energy, and reduced water contact angles in Topas 112/011. In GO, irradiation preferentially removes oxygen, partially restores sp^2^ domains at low fluences, and generates defects and lattice disorder at higher fluences. AFM measurements indicate a decrease in surface roughness in polymers and a moderate evolution in GO. Sheet resistivity decreases in all materials, with Topas showing moderate enhancement and GO a relatively monotonic reduction.

## Figures and Tables

**Table 1:** SRIM-calculated S_e_, S_n_, R_p_, and ΔR_P_ values of 1 MeV Au ions for Topas 112/011 and GO.

Samples	*S*_e_ [eV/Å]	*S*_n_ [eV/Å]	*S*_e_/*S*_n_	*R*_p_ [nm]	Δ*R*_P_ [nm]
TOPAS 112	1.228 × 10^2^	1.600 × 10^2^	0.77	422	51
TOPAS011	1.223 × 10^2^	1.603 × 10^2^	0.76	421	51
GO	1.121 × 10^2^	1.833 × 10^2^	0.61	358	51

**Table 2:** The results of the RBS and ERDA measurements. The elemental composition of TOPAS 112/011 and GO foils irradiated by 1 MeV Au ions with fluences in the range of 1 × 10^14^–2.5 × 10^15^ cm^−2^.

Material	Ion Fluence [Au/cm^2^]	C [at. %]	O [at. %]	H [at. %]	N [at. %]	C/O	C/H
Topas 112	pristine	53.1	0.9	46.0		59.0	1.2
	1 × 10^14^	62.0	6.0	32.0		10.3	1.9
	1 × 10^15^	78.9	0.2	21.0		394.5	3.8
	2.5 × 10^15^	79.9	0.2	20.0		399.5	4.0
Topas 011	pristine	53.0	1.5	45.5		35.3	1.2
	1 × 10^14^	57.1	8.9	34.0		6.4	1.7
	1 × 10^15^	72.7	0.8	26.5		90.9	2.7
	2.5 × 10^15^	83.0	1.2	15.8		69.2	5.3
GO *	pristine	64.9	18.1	13.0	4.0	3.6	5.0
	1 × 10^14^	71.6	15.0	9.6	3.8	4.8	7.5
	1 × 10^15^	77.2	11.0	8.0	3.8	7.0	9.7
	2.5 × 10^15^	81.6	6.0	7.8	3.6	13.6	10.5

* Elements such as Mn, K, or S were also present in the GO in concentrations below 1%; however, these were omitted from the Table to facilitate a clearer comparison of the primary components.

**Table 3:** R_a_ and RMS of TOPAS 112 and 011 and GO foils irradiated by 1 MeV Au ions with fluences in the range of 1 × 10^14^–2.5 × 10^15^ cm^−2^.

Material	Ion Fluence [Au/cm^2^]	Ra [nm]	RMS [nm]
Topas 112	pristine	1.20 ± 0.28	1.35 ± 0.38
	1 × 10^14^	0.30 ± 0.06	0.39 ± 0.10
	1 × 10^15^	0.71 ± 0.17	1.07 ± 0.70
	2.5 × 10^15^	0.75 ± 0.19	1.12 ± 0.35
Topas 011	pristine	1.16 ± 0.27	1.45 ± 0.40
	1 × 10^14^	0.72 ± 0.19	1.03 ± 0.26
	1 × 10^15^	1.05 ± 0.18	1.51 ± 0.63
	2.5 × 10^15^	1.24 ± 0.41	1.79 ± 0.71
GO	pristine	19.93 ± 4.07	23.88 ± 4.32
	1 × 10^14^	26.34 ± 9.07	32.91 ± 13.37
	1 × 10^15^	24.85 ± 2.58	28.91 ± 2.34
	2.5 × 10^15^	16.44 ± 4.85	19.89 ± 5.52

**Table 4:** The XPS measurement of Topas 112/011 and GO foils irradiated by 1 MeV Au ions with fluences in the range of 1 × 10^14^–2.5 × 10^15^ cm^−2^.

Material	Ion Fluence [Au/cm^2^]	C–C/C–H (284.8 eV)	C–O (286.1–286.6 eV)	C=O (287.3–288 eV)	O–C=O (288.6–289.2 eV)	π–π*	C/O
Topas 112	pristine	100					
	1 × 10^14^	72 ± 3	17 ± 3	5 ± 2	3 ± 1	2 ± 1	17
	1 × 10^15^	76 ± 3	12 ± 3	7 ± 2	3 ± 1	2 ± 1	3
	2.5 × 10^15^	79 ± 3	11 ± 3	3 ± 1	3 ± 1	4 ± 1	19
Topas 011	pristine	100					
	1 × 10^14^	74 ± 3	13 ± 3	4 ± 2	6 ± 1	3 ± 1	13
	1 × 10^15^	76 ± 3	12 ± 3	5 ± 2	5 ± 1	2 ± 1	9
	2.5 × 10^15^	81 ± 3	10 ± 3	3 ± 2	3 ± 1	3 ± 1	12
GO	pristine	66 ± 3	17 ± 3	9 ± 2	5 ± 2	3 ± 1	4
	1 × 10^14^	74 ± 3	13 ± 3	5 ± 2	5 ± 1	3 ± 1	5
	1 × 10^15^	80 ± 3	10 ± 3	4 ± 1	4 ± 1	2 ± 1	7
	2.5 × 10^15^	78 ± 3	13 ± 3	4 ± 2	3 ± 1	2 ± 1	7

**Table 5:** Calculated SFE with disperse and polar components of TOPAS 112 and 011 and GO foils irradiated by 1 MeV Au ions with fluences in the range of 1 × 10^14^–2.5 × 10^15^ cm^−2^.

Material	Ion Fluence [Au/cm^2^]	OWRK SFE [mN/m]	Disperse [mN/m]	Polar [mN/m]
Topas 112	pristine	33.74 ± 3.00	33.60 ± 2.86	0.14 ± 0.14
	1 × 10^14^	40.83 ± 1.01	38.06 ± 0.77	2.77 ± 0.25
	1 × 10^15^	38.72 ± 1.31	35.94 ± 1.00	2.78 ± 0.31
	2.5 × 10^15^	39.59 ± 1.41	35.92 ± 0.94	3.67 ± 0.47
Topas 011	pristine	32.88 ± 3.01	32.43 ± 2.67	0.45 ± 0.34
	1 × 10^14^	38.98 ± 2.00	36.72 ± 1.16	2.25 ± 0.85
	1 × 10^15^	40.00 ± 1.05	37.04 ± 0.70	2.95 ± 0.34
	2.5 × 10^15^	40.12 ± 1.11	36.41 ± 0.50	3.71 ± 0.61
GO	pristine	55.06 ± 7.54	39.20 ± 2.90	15.86 ± 4.64
	1 × 10^14^	47.40 ± 6.39	41.25 ± 1.26	6.16 ± 5.12
	1 × 10^15^	45.81 ± 4.70	39.82 ± 1.33	5.99 ± 3.37
	2.5 × 10^15^	44.77 ± 1.72	41.64 ± 1.36	3.13 ± 0.36

## Data Availability

All data for this paper are mentioned in Zenodo https://doi.org/10.5281/zenodo.18445011.
